# Cervical epidural hematoma after spinal manipulation therapy: a case report

**DOI:** 10.1186/s12891-019-2871-y

**Published:** 2019-10-22

**Authors:** Qian Chen, Jun-fei Feng, Xin Tang, Yu-ling Li, Lu Chen, Guo Chen

**Affiliations:** 1 Department of Orthopaedic Surgery, the Affiliated Hospital of North Sichuan Medical College, Nanchong, 637000 Sichuan Province China; 2 Zunyi Medical University, Zunyi, 563000 Guizhou Province China; 3 Sichuan Provincial Orthopedic Hospital, Chengdu, 610041 Sichuan China

**Keywords:** Cervical epidural hematoma, Spinal manipulation, Spinal cord injury

## Abstract

**Background:**

Cervical spinal manipulation therapy is a common non-invasive treatment for neck pain and stiffness, and has been widely used in the population. However, most people do not pay attention to the potential risks of neck manipulation, such as ligament damage, fractures, and spinal cord injuries. Epidural hematoma is a disease in which blood accumulates in the epidural space of the vertebral body. This disease is usually caused by trauma or iatrogenic surgery, and may be associated with blood coagulopathies, neoplasms, or degenerative spinal disease. Reports of epidural hematoma caused by cervical spinal manipulation are rare.

**Case presentation:**

We present the case of a patient with tetraplegia and spinal shock after neck manipulation. A physical examination of the patient on admission found tenderness in the neck and increased muscle tension in both upper limbs. The superficial sensation of the upper limb disappeared, but the deep sensation still remained. The lower extremity had 0/5 power on both sides. The sensation below the T2 level completely disappeared. A cervical magnetic resonance imaging scan showed an acute posterior epidural hematoma from the C3–T3 vertebrae. Ultimately, the patient underwent emergency hematoma removal and showed partial improvement in symptoms of paralysis during follow-up.

**Conclusions:**

Although spinal manipulation is simple and neck pain is common and recurrent in the general population, the basic condition and disease history of patients should be determined before manipulation. For high-risk patients, caution should be applied for cervical spinal manipulation or it should be prohibited. For a suspected hematoma, MRI should be used at an early stage to diagnose and locate the hematoma.

## Background

Cervical spinal manipulation therapy is widely used among the people, and it is part of the complementary treatment for neck pain and stiffness. There are many medical institutions and non-medical institutions that offer various types of manipulation. Although neck manipulation is simple, the serious complications caused by neck manipulation cannot be ignored, such as vertebral artery dissection, spinal cord injury, cervical subluxation, and cerebrovascular accidents [1, 2]. Cervical epidural hematoma is a serious complication after neck spinal manipulation and is rare. Few cases of severe quadriplegia caused by neck manipulation have been reported [3–12]. Most of these cases suffered from various underlying diseases, such as coagulation dysfunction and cervical vascular malformations. We report a patient with tetraplegia due to neck manipulation. Our patient is unique because he had no history of these diseases or medication use.

## Case presentation

**Fig. 1:**
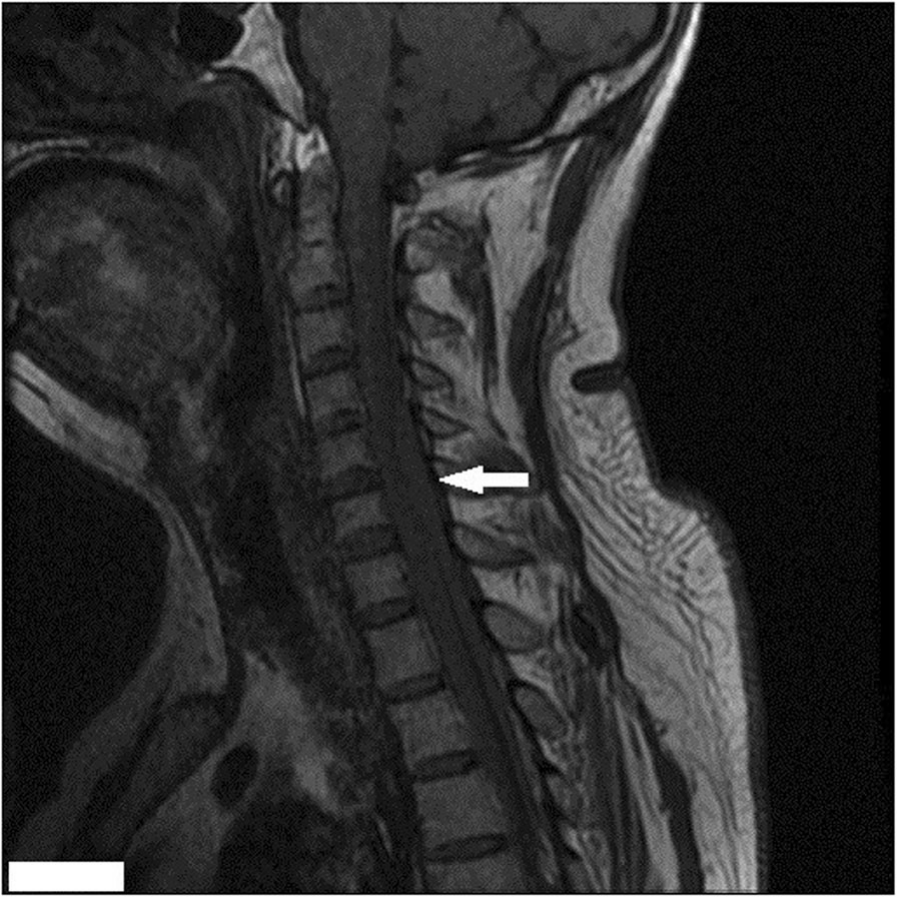
Sagittal MRI of the cervical spine (T1-weighted image). Hypointense collection over the posterior aspect of the spinal cord with cord edema can be seen

**Fig. 2:**
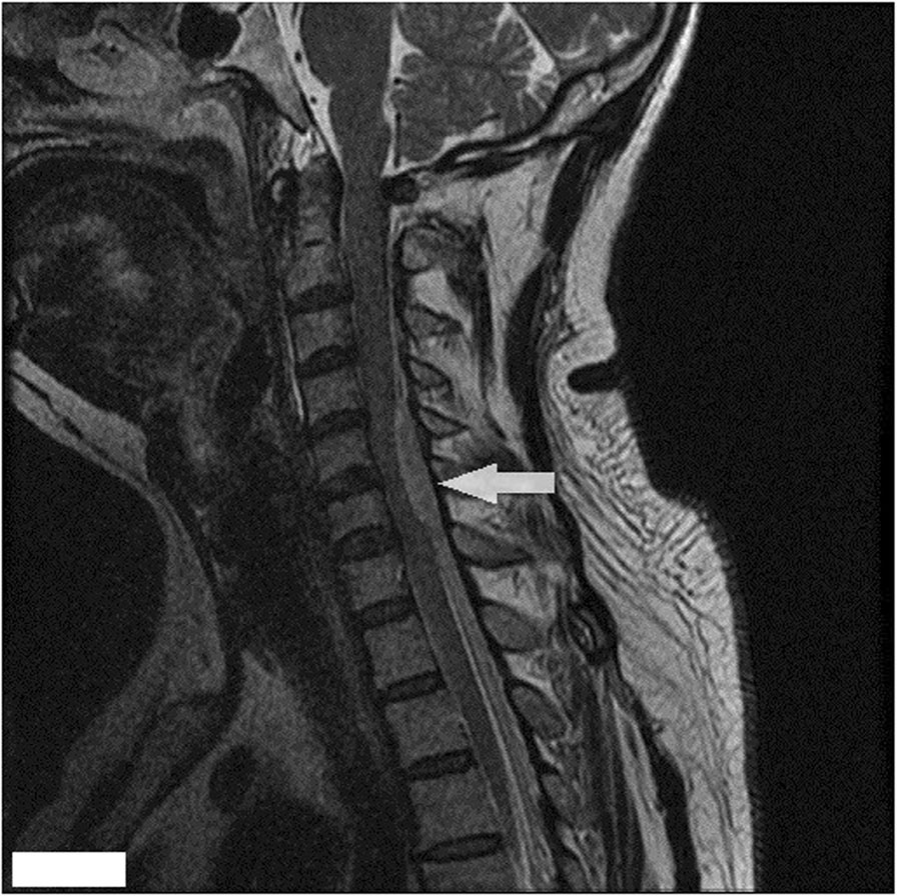
Sagittal MRI of the cervical spine (T2-weighted image). An area of increased intensity from the C3–C7 levels can be seen

**Fig. 3:**
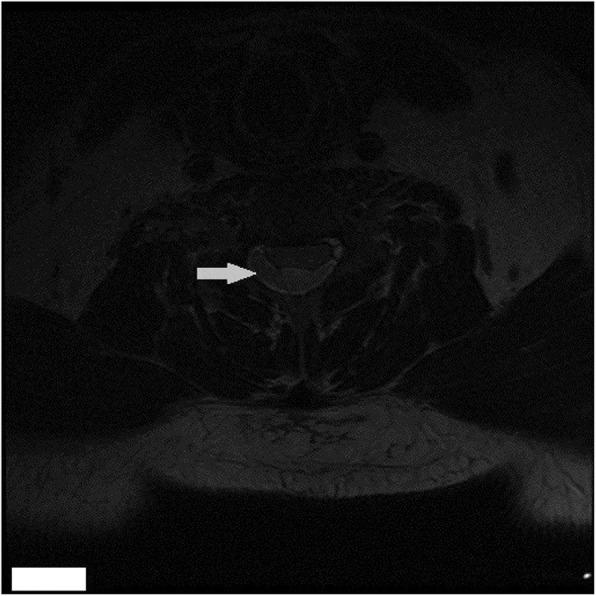
Transaxial MRI of the cervical spine (T2-weighted image). Compression over the right posterolateral aspect of the spinal cord at the C5/C6 level can be seen

**Fig. 4:**
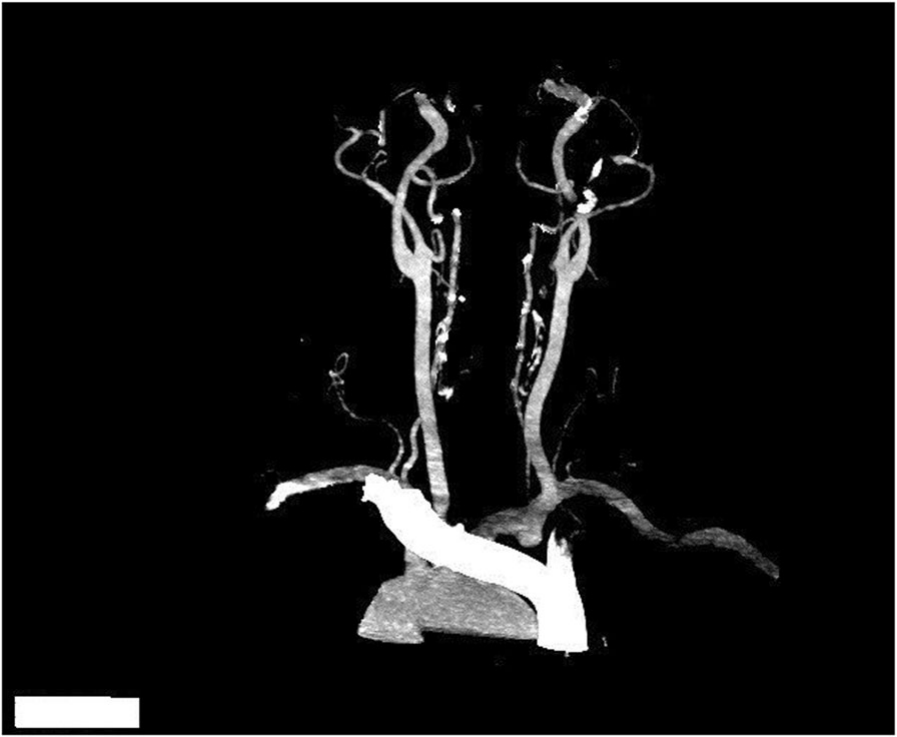
CT angiography of neck. CT angiography of the neck shows that there is no malformation of neck vessels

We experienced a 55-year-old man who developed tetraplegia after neck spinal manipulation for stiff neck pain. The patient was healthy with no significant medical history and no previous history of taking medication (the patient denied taking aspirin or any other anticoagulant medications). But he had several previous mild neck pains and was relieved after manipulation treatment. The patient was treated with cervical manipulation and he felt pain and numbness in his lower limbs about 2 h after the end of the manipulation. The symptoms of his sensory abnormalities gradually worsened and spread upwards. The patient felt seriously ill and was immediately sent by his relatives to the emergency room of our hospital to visit a doctor. After being admitted to the emergency room, the patient was mentally alert during a physical examination. There was tenderness in the neck and increased muscle tension in both upper limbs. The superficial sensation of the upper limb had disappeared, but the deep sensation still remained. The lower extremity had 0/5 power on both sides. There were findings of sensory deprivation at the T2 dermatome and below, and anal tone was absent with the bulbocavernosus reflex. An imaging examination was performed after an indwelling catheter was inserted. There were no abnormal findings on a cervical vertebral X-ray and brain computed tomographic (CT) scans. A cervical magnetic resonance imaging (MRI) scan showed an acute posterior epidural hematoma from the C3 to T3 vertebrae (Figs. 1 and 2). MRI also showed a large heterogeneous collection within the right lateral epidural space of C4 until T1, which was consistent with the hyperacute epidural hematoma, with cord edema at the same level (Fig. 3). The hematoma resulted in spinal stenosis, the narrowest of which was located at the C5 and C6 levels. There was no evidence of vertebral body fracture or subluxation. Clinical laboratory results at admission were normal and blood investigations showed that platelet counts were within the normal range with a normal coagulation profile (Table 1). Subsequently, to exclude vascular malformations, the patient was scheduled to undergo CT angiography. CT angiography showed no malformation of the neck vessels (Fig. 4).

**Table 1:** Clinical laboratory coagulation test results

Inspection item	Quantitative results	Reference value
PT	12.700	12.40–14.5
INR	0.9800	0.90–1.15
PT%	103.00	78.0–124.0
APTT	30.40	28.0–45.0
TT	15.20	15.0–19.0

We started intravenous prednisolone infusion to the patient to alleviate spinal edema and to prepare for spinal canal decompression and evacuation of the hematoma. During the operation, we saw a C4–T1 epidural hematoma and compression of the spinal cord at the corresponding level, especially at the C5–C6 levels. The hematoma was located in the right posterior part of the spinal cord, which is consistent with the results of MRI. The hematoma began to thin below the T1 level, and spinal cord compression was reduced accordingly. Therefore, we only removed the hematoma at the C4–T1 levels.

After surgery, the patient was sent to the intensive care unit for further treatment. On postoperative day 1, there was some improvement in neurology, with a returning of upper extremity strength from 0/5 to 2/5. Superficial sensation and deep sensation between the T2–T8 levels changed from deprivation to hypoesthesia. On the 7th day after the operation, the patient’s symptoms improved further. His upper limb muscle strength increased to 4/5, but sensation below the T10 level was still absent. This condition remained until he was discharged from hospital. During a follow-up of 3 months after discharge, neurological function of the patient did not greatly recover.

## Discussion and conclusion

Cervical spinal manipulation is a common practice, which is performed by either professional or non-professional personnel, owing to its relatively few invasive treatments. It is defined by the International Federation of Orthopedic Manipulative Physical Therapists (IFOMPT) as: “A passive, high velocity, low amplitude thrust applied to a joint complex within its anatomical limit with the intent to restore optimal motion, function, and/ or to reduce pain” [13]. The known complications resulting from cervical spinal manipulation include cerebral stroke from local pressure on the blood vessels, ligament injury or fractures from excessive pressure or rotation, and spinal cord injury [4, 14]. After cervical spine manipulation, the estimated incidence of serious adverse events ranges from 1 per 50,000 to 1 per 5.85 million manipulations [2]. According to reports that the likelihood of injury following spinal manipulation was increased among patients with a chronic coagulation defect, inflammatory spondylopathy, osteoporosis, aortic aneurysm and dissection, or long-term use of anticoagulant therapy [15]. Epidural hematoma is a disease in which blood accumulates in the epidural space of the vertebral body. This disease is usually caused by trauma or iatrogenic surgery, and may be associated with blood coagulopathies, neoplasms, or degenerative spinal disease. Reports of epidural hematoma caused by neck manipulation are rare, with less than 10 cases reported [3–10]. Most cases occurred in the cervical spine, not in the thoracic or lumbar spine. Some of these cases had cervical spondylosis or a history of oral anticoagulants. Table 2 summarizes the cases of epidural hematoma after neck manipulation [3–12]. In most cases, the location of the hematoma was either posterior or posterolateral. The hematoma in our case was located at the right posterior side. The pathological mechanism of spinal epidural hematoma remains unclear. The mechanism of spinal epidural hematoma might be the same as that of intracranial epidural hematoma [16]. However, some researchers believe that spinal epidural hematoma is caused by injury of the epidural venous plexus or a sudden increase of venous pressure [16]. The incidence of spinal epidural hematoma is higher in patients with coagulation disorders and in those taking anticoagulants [17]. In a case reported by Whedon et al. [5], the patient had to take coumarin for a long time because of atrial fibrillation and showed stiffness after neck manipulation. Subsequent laboratory tests showed abnormal coagulation function. The results of coagulation-related examinations in this case were normal and there was no history of taking anticoagulants (Table 1). Heiner [9] reported another interesting case in which the patient did not have the above-mentioned risk factors. However, the patient was pregnant at that time. Because of the change in venodynamics and a decrease in venous pressure in the epidural space relative to venous pressure, the pressure gradient of epidural vessels increased, which easily led to epidural hematoma [18]. We observed that most of these cases report scarcely description of the clinical characteristics which are possible risk factors for serious complications in patients, such as smoking, cervical trauma, recent infection, hypertension, etc. (This is where our cases are limited) It could be that the manipulating professionals did not see the need to report or were unaware of these items or were more focused on the treatment strategy and recovery after hospitalization [13]. The CARE statement was published to guide transparency and accuracy of case reports as well as to improve the quality of case reports [19, 20].

**Table 2:** Summary of reported cases of cervical epidural hematoma after spinal manipulation therapy

Reference	Age (years)	Gender	Symptoms	Level	Location of hematoma	Treatment	Outcome
Segal et al. 1996 [3]	33	Female	Paraplegia	C4–6	posterior	Surgery	
Tseng et al.,2002 [6]	67	Female	Hemiparesis	C3–5	posterolateral	Surgery	Recovery
Saxler G et al.,2002 [4]	27	Female	Headache	C1-S1	not reported	Conservative	Recovery
Whedon et al.,2006 [5]	79	Male	Lower extremity paralysis	C2–4	posterolateral	Surgery	Recovery
Domenic- cci et al.,2007 [8]	52	Female	Hemiparesis	C4-T1	posterolateral	Surgery	Recovery
Heiner et al.,2009 [9]	38	Female	Upper extremity paralysis	C4	posterolateral	Conservative	Recovery
Meng et al.,2015 [11]	40	Male	Upper extremity paralysis	C2-T2	posterolateral	Surgery	Recovery
Fattahi et al.,2017 [12]	44	Female	Tetraplegia	C1–4	anterior	Conservative	Recovery
Ling et al.,2017 [7]	33	Male	Tetraplegia	C4–7	posterolateral	Surgery	Die
Ryu et al.,2018 [10]	38	Male	Paraparesis	C6-T1	anterior	Conservative	Recovery
Present case	55	Male	Tetraplegia	C3-T3	posterolateral	Surgery	Partial recovery

*C* cervical, *T* thoracic, *S* sacrum

Cervical spinal epidural hematoma is usually characterized by neck pain, scapular pain, and varying degrees of neurological deficits [5]. An early MRI scan is necessary for this type of patient, and it can accurately determine the location and severity of the hematoma. Patients with mild neurological symptoms and a stable condition can be treated conservatively. In a case reported by Ryu et al. [10], because the patient’s symptoms rapidly improved, no surgical treatment was required and he was discharged in only 1 week. Surgical treatment should be performed in patients with severe neurological deficits or progressive severe symptoms. In a case reported by Ling et al. [7], surgery was performed after the patient was admitted for tetraplegia after neck manipulation. These authors believed an earlier surgical intervention would have delivered a better outcome and improvement. Our patient who had a severe nerve defect underwent surgical treatment and achieved good results. Surgery prevented further compression and edema of the cervical spinal cord, which created a favorable environment for subsequent recovery of nervous function.

How to improve the safety of cervical spine manipulation? It is important that every potential serious adverse event caused by vascular or other pathologies should be prevented. Thus, thorough patient interviewing, clinical assessment, interpretation and analysis are significant components needed to define an indication for cervical spine manipulation [21]. Table 3 presents a summary of contraindications and precautions for cervical spine manipulation [21]. Cervical spine manipulation should not be performed when contraindications are present [17]. Prior to manipulation, a risk-benefit analysis should be performed and that includes the following three steps [21] :①identifying a possible vasculogenic contribution or other serious pathology; ②determining whether there is an indication or contraindication for mobilization or manipulation; ③sessing the presence of any potential risk factors associated with potential serious adverse events which are reported to occur after cervical spine mobilization and/or manipulation. Potential risk factors, risk signals and contraindications can be found in interviews with patients and this information can provide a basis to create initial hypotheses to be further investigated in the clinical examination [22]. Physical examination before manipulation is also necessary, because the examination of abnormal sensory and muscular strength of limbs maybe occur in patients with cervical epidural hematoma and a positive test can be regarded as an indicator of the patient’s risk of getting severe complications during a cervical manipulation. Such as spinal epidural hematoma can present with features ranging from simple pain with radiculopathy to complete paraplegia or quadriplegia [23]. If we just adopt spinal manipulation because of stiffness and pain in the neck and ignore the abnormal results of other physical examinations, it may lead to serious consequences. The upper cervical spine instability tests and premanipulative vertebrobasilar insufficiency tests these tests can be valuable in detecting upper cervical spine instability or vertebrobasilar insufficiency, but their applicability as primary screening test has yet to be confirmed [24, 25]. Moreover, cervical manipulation should not be performed at the end of range of cervical movement, particularly extension and rotation [22].

**Table 3:** Contraindications and precautions to perform cervical spinal manipulation [21]

Contraindications	Precautions
(Acute) fracture	Inflammatory disease
Relevant recent trauma	Rheumatoid arthritis
Dislocation	Ankylosing spondylitis
Ligamentous rupture	History of cancer
Instability	Long-term steroid use
Active cancer	Osteoporosis
Acute myelopathy	Systemically unwell
Spinal cord damage	Hypermobility syndromes
Upper motor neuron lesions	Connective tissue disease
Multi-level nerve root pathology	A first sudden episode before age 18 or after age 55
Worsening neurological function	Cervical anomalies
Recent surgery	Local infection
Acute soft tissue injury	Throat infection
Unremitting, severe, non-mechanical pain	Recent manipulation by another health professional
Unremitting night pain	Vascular disease
Vertebral / carotid artery abnormalities	Blood clotting disorders / alterations in blood properties
Vertebrobasilar insufficiency	Anticoagulant therapy
	Absence of a plausible mechanical explanation for the patient’s symptoms
	Immediately post-partum

In conclusion, neck pain is common and recurrent in the general population, but in the absence of neurological signs and symptoms, there is no practical, clinically valid screening tests to identify underlying risks in patients with neck pain. So, history taking and patient characteristics are very important. Patients with a suspected hematoma should first be examined by MRI to make a definite diagnosis and guide further treatment.

## Data Availability

All data analyzed during this study are included within the manuscript. The datasets used and/or analyzed during this study are available from the first author on reasonable request.

## Abbreviations

APTT: Activated partial thromboplastin time
CT: Computed tomography
INR: International normalized ratio
MRI: Magnetic resonance imaging
PT: Prothrombin time
TT: Thrombin time
